# Difference between emergent aquatic and terrestrial monocotyledonous herbs in relation to the coordination of leaf stomata with vein traits

**DOI:** 10.1093/aobpla/plaa047

**Published:** 2020-09-11

**Authors:** Wanli Zhao, Peili Fu, Guolan Liu, Ping Zhao

**Affiliations:** 1 Key Laboratory of Vegetation Restoration and Management of Degraded Ecosystems, South China Botanical Garden, Chinese Academy of Sciences, Guangzhou, China; 2 Center of Plant Ecology, Core Botanical Gardens, Chinese Academy of Sciences, Guangzhou, China; 3 Guangdong Provincial Key Laboratory of Applied Botany, South China Botanical Garden, Chinese Academy of Sciences, Guangzhou, China; 4 Shandong Key Laboratory of Eco-Environmental Science for Yellow River Delta, Binzhou University, Binzhou, China; 5 CAS Key Laboratory of Tropical Forest Ecology, Xishuangbanna Tropical Botanical Garden, Chinese Academy of Sciences, Yunnan 666303, China; 6 Ailaoshan Station of Subtropical Forest Ecosystem Studies, Xishuangbanna Tropical Botanical Garden, Chinese Academy of Sciences, Jingdong, Yunnan 676209, China

**Keywords:** Leaf hydraulics, optimization, Poaceae, stomatal density, vein density

## Abstract

Emergent aquatic plants mostly occur in shallow waters and root in bottom substrates, but their leaves emerge from the water surface and are thus exposed to air, similar to the leaves of terrestrial plants. Previous studies have found coordination between leaf water supply and demand in terrestrial plants; however, whether such a coordination exists in emergent aquatic plants remains unknown. In this study, we analysed leaf veins and stomatal characteristics of 14 emergent aquatic and 13 terrestrial monocotyledonous herb species (EMH and TMH), with 5 EMH and 8 TMH belonging to Poaceae. We found that EMH had significantly higher mean leaf area, leaf thickness, stomatal density, stomatal number per vein length and major vein diameter, but lower mean major vein length per area (VLA) and total VLA than TMH. There was no significant difference in stomatal length, minor VLA and minor vein diameter between the two groups. Stomatal density and total VLA were positively correlated among the EMH, TMH, as well as the 8 Poaceae TMH species, but this correlation became non-significant when data from both the groups were pooled. Our results showed that the differences in water supply between emergent aquatic and terrestrial plants modify the coordination of their leaf veins and stomatal traits.

## Teaser

We analysed leaf veins and stomatal characteristics of 14 emergent aquatic and 13 terrestrial monocotyledonous herb species. We found that emergent aquatic species had significantly higher stomatal density and stomatal number per vein length, but lower mean major vein length per area (VLA) and total VLA than terrestrial species. Stomatal density and total VLA were positively correlated among the emergent aquatic species and terrestrial species, but this correlation became non-significant when data from the two groups were pooled. Our results showed that the differences in water supply between emergent aquatic and terrestrial plants modify the coordination of their leaf veins and stomatal traits.

## Introduction

In leaves, the xylem supplies water to the photosynthetic tissues to prevent their desiccation during photosynthetic CO_2_ exchange with the atmosphere (Brodribb *et al.* 2007; Feild and Brodribb 2013). Stomata control gas exchange between the leaves and atmosphere (Hetherington and Woodward 2003; Simonin and Roddy 2018). Therefore, both stomatal density and size play vital roles in controlling maximum transpiration, i.e. leaf water demand (Franks and Beerling 2009). Leaf veins transport water from the petiole across the lamina to the mesophyll mainly for transpiration (Niklas 1999). Indeed, previous studies have shown that vein density (vein length per leaf area, VLA) is a key determinant of leaf water supply capacity in terrestrial plants (Sack and Scoffoni 2013; Scoffoni and Sack 2017). Coordination between stomatal density and VLA across species indicates different strategies for the maintenance of water balance (Brodribb *et al.* 2013; Schneider *et al.* 2017). A positive correlation between the minor VLA and stomatal density has been found in many species across different habitats (Zhang *et al.* 2012; Carins Murphy *et al.* 2016; Zhao *et al.* 2016). However, we still know little about this relationship in aquatic plants.

Although many species show coordination between stomatal density and VLA, some species have unique strategies for maintaining water balance. For example, no significant positive correlation had been found between VLA and stomatal density among terrestrial and epiphytic *Cymbidium* species, which is mainly due to the high water storage capacity of these species (Zhang *et al.* 2015). The high capacitance buffers water potential declines in the transpiration stream, and then reduces the dependence of transpiration on water uptake from the soil (Meinzer and Grantz 1991; Ogburn and Edwards 2013; Roddy *et al.* 2018). Available water resources play important roles in leaf venation development (Uhl and Mosbrugger 1999; Roddy *et al.* 2019), and a negative correlation has been found between VLA and water availability in several herbs (Napp-Zinn 1988). Whereas a positive correlation between these factors has been found in plants growing under nearly saturated air humidity in tropical rainforests (Pyykkö 1979). Under arid conditions, some species have apparent over-investment in leaf venation to compensate for the adverse effect that the thicker leaves have on photosynthesis (de Boer *et al.* 2016). In contrast, plants growing in water may have lower drought stress given their submergence in water, whereas exposure of their leaves to air may render a high vapour pressure deficit that may influence the evolution of vein and stomatal traits (Fanourakis *et al.* 2011; Hovenden *et al.* 2012; Carins Murphy *et al*. 2014).

The coordination between VLA and stomatal density in maintaining homeostasis in leaf water content is crucial for continued physiological function (Brodribb *et al.* 2011; Roddy *et al.* 2020). Zhao *et al.* (2016) found that tree species in subtropical mountain forest had lower VLA, but similar stomatal density when compared with tree species in a tropical mountain forest, which caused significant differences in the coordination between stomatal density and VLA between these two types of forests. The stomatal number per vein length, which is calculated from dividing stomatal density by VLA (Zhao *et al.* 2017), could also be used to compare the difference in the coordination between leaf water supply and demand. Zhao *et al.* (2017) found that three leguminous species under certain environmental conditions had stable stomatal number per vein length, which showed the coordination between leaf water supply and demand. When the environmental conditions changed, the stomatal number per vein length would change accordingly. At present, studies comparing the differences between stomatal number per vein length and VLA and stomatal density are rare.

Emergent aquatic plants mostly occur in shallow waters and root in the bottom substrates, but their leaves emerge from the water surface and are thus exposed to air, which are similar to terrestrial plant leaves (Golub *et al.* 1991; Lacoul and Freedman 2006). Approximately 11 % of monocotyledonous plants are aquatic (Les and Schneider 1995; Lacoul and Freedman 2006; Conklin *et al.* 2019). Most monocots have a distinct hierarchy of gridded ‘parallel’ or ‘striate’ major veins with midribs, large and intermediate longitudinal veins that are analogous to major vein orders and small longitudinal veins and transverse veins that are analogous to minor veins (Ueno *et al.* 2006; Sack and Scoffoni 2013). The pan-tropical Ochnaceae species have dense major veins, whereas the coordination of total VLA and stomatal density across 55 species in this family is maintained (Schneider *et al.* 2017). The dense major veins also exist in emergent aquatic monocotyledonous herbs (EMH), while the relationship between the total VLA and stomatal density of those species is still unclear.

Noticeably, water supply is not a limiting factor for EMH, but it is for terrestrial monocotyledonous herbs (TMH), especially in the dry season. With sufficient water supply, plants typically have higher photosynthesis and transpiration rates (Passioura 2002). Under sufficient water supply, a low VLA has the potential benefits of reducing construction costs and displacing mesophyll (Baresch *et al.* 2019; Sporck and Sack 2010). In this study, we chose EMH as the subject and TMH as the baseline to clarify the relationship between VLA and stomatal density in these two groups. We hypothesized that compared with terrestrial species, the emergent aquatic species would have higher vein diameter but lower VLA, and higher stomatal number per vein length. The results of this study may deepen the understanding of the relationship between leaf vein and stomatal traits.

## Methods

### Site and sampling

This study was carried out in the South China Botanical Garden (SCBG; 23°10′N, 113°21′E, elevation 41 m), Chinese Academy of Sciences, Guangzhou City, Guangdong Province, China. The mean annual temperature in the garden is 21.7 °C and the mean annual precipitation is 1761 mm (with more than 80 % rain from May to September).

**Figure 1.**
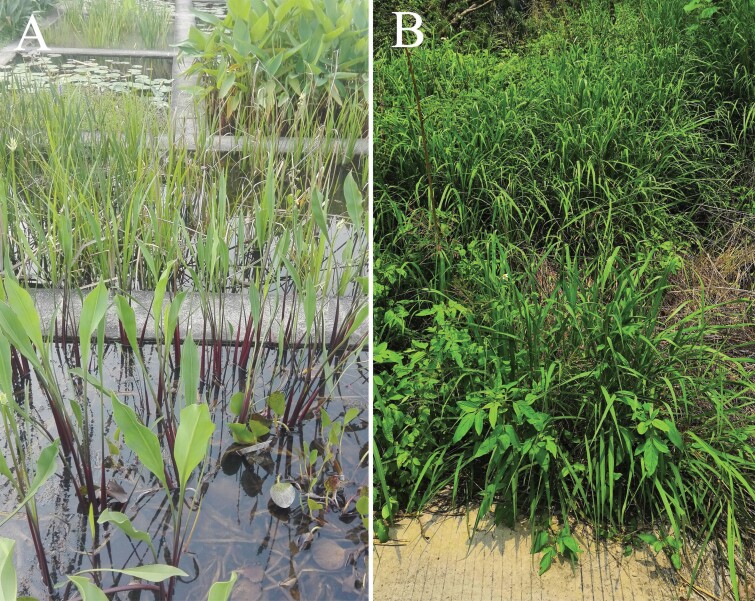
Habitat of emergent aquatic (A) and terrestrial (B) herbs. Photos by WL Zhao and QG Mao.

EMH grow in a shallow freshwater pool at SCBG (Fig. 1) and TMH are common and grow along the roads. We collected 4–6 mature leaves from 4 to 6 individual plants in full sunlight of each species and stored them in a refrigerator at 4 °C in July 2017. In total, 14 EMH, belonging to eight families and 13 TMH belonging to six families, were collected (Table 1). As 5 EMH and 8 TMH of the 27 species in this study belonged to Poaceae (EMH_p_ and TMH_p_, respectively), we also checked our hypothesis at the family level, which would reduce the influence of phylogenetic factors.

**Table 1. The:** code, Latin names, Family, photosynthetic pathway as well as leaf vein type of the 14 emergent aquatic and 13 terrestrial monocotyledonous herbs (EMH and TMH) species. RV = reticulate venation, PV = parallel venation.

code	Species	Family	C3/C4	Leaf vein type
	EMH			
1	*Alisma canaliculatum*	Alismataceae	C3	RV
2	*Arundo donax*	Poaceae	C3	PV
3	*Canna glauca*	Cannaceae	C3	RV
4	*Canna indica*	Cannaceae	C3	RV
5	*Cortaderia selloana*	Poaceae	C3	PV
6	*Cyperus alternifolius*	Cyperaceae	C4	PV
7	*Limnocharis flava*	Alismataceae	C3	RV
8	*Oryza rufipogon*	Poaceae	C3	PV
9	*Oryza sativa*	Poaceae	C3	PV
10	*Phragmites australis*	Poaceae	C4	PV
11	*Pontederia cordata*	Pontederiaceae	C3	RV
12	*Thalia dealbata*	Marantaceae	C3	PV
13	*Typha angustifolia*	Typhaceae	C3	PV
14	*Typha orientalis*	Typhaceae	C3	PV
	TMH			
15	*Alpinia japonica*	Zingiberaceae	C3	PV
16	*Axonopus compressus*	Poaceae	C4	PV
17	*Coix lacrymajobi*	Poaceae	C4	PV
18	*Commelina communis*	Commelinaceae	C3	RV
19	*Cordyline fruticosa*	Asparagaceae	C3	PV
20	*Cyperus rotundus*	Cyperaceae	C4	PV
21	*Digitaria sanguinalis*	Poaceae	C4	PV
22	*Fargesia spathacea*	Poaceae	C3	PV
23	*Imperata cylindrica*	Poaceae	C4	PV
24	*Maranta arundinacea*	Marantaceae	C3	PV
25	*Oplismenus undulatifolius*	Poaceae	C3	PV
26	*Panicum bisulcatum*	Poaceae	C4	PV
27	*Setaria viridis*	Poaceae	C4	PV

The leaves were scanned using an HP CLJM277 scanner, and leaf area was measured using Image J software (http://rsbweb.nih.gov/ij/index.html).

### Stomatal density and modelled maximum stomatal conductance

As all the species in this study were amphistomatous, stomatal density, stomatal length and width (refer to guard cell length and width, respectively) were determined from both adaxial and abaxial cuticles of EMH and TMH. The leaves were prepared and measured following the protocols of Zhao *et al.* (2016). Because of stomatal size–number trade-off, we also estimated the theoretical modelled maximum stomatal conductance (*g*_max_) as reported by Franks and Farquhar (2001):


$$\begin{array}{l} g_{max}=\frac{d}{v}\times D\times \frac{a}{l+(\pi /2)\sqrt{a/\pi }}, \end{array}$$ (1)

where *d* is the diffusivity of water in air (m^2^ s^−1^); *υ* is the molar volume of air (m^3^ mol^−1^); *D* is the stomatal density; *a* is the maximum pore area approximated as π(*p*/2)^2^, where *p* is 1/2 guard cell length; *l* is the pore depth that is represented by 1/2 guard cell width, assuming guard cells inflate to a circular cross-section (Franks and Beerling 2009).

### Leaf vein measurements and categories

The leaves that had been used to measure stomatal traits were also used to measure the VLA. The leaves were placed in bottles containing 5 % NaOH aqueous solution and were heated in a water bath (Yiheng HWS24, Shanghai, China) until the veins were exposed. We used distilled water to soak the leaves for 30 min, and then the leaves were dyed with 1 % methylene blue solution, rinsed again, mounted on slides and photographed. For the species with reticulate pattern veins, we distinguished vein order hierarchy according to Sack *et al.* (2012), and for other species with parallel or striate venation, we distinguished vein order hierarchy according to Christin *et al.* (2013). Image J (http://rsbweb.nih.gov/ij/index.html) was employed to measure the VLA of different vein categories. Although the transverse veins of parallel or striate venation have an important role in leaves, the proportion of them was small in the whole leaf venation (McKown and Dengler 2010; Lundgren *et al.* 2019), and we excluded them in this study. We measured major VLA (1°–3°) separately, but for minor VLA, 4° and higher orders were grouped into one class. The major vein diameters were performed including the bundle sheath, and we measured the different orders from the middle of the leaves, and the mean minor vein diameter was calculated for orders 4° and higher. Stomata number per vein length (no. mm^-1^) was calculated from dividing stomatal density by total VLA.

We roughly estimated the xylem construction cost of leaf veins with a dimensionless index of cell wall volume per leaf area (CC, McKown *et al.* 2010). A modified, yet simplified, method of Schneider *et al.* (2017) for lumen diameter and conduit density per vein order determination was applied for total vein diameter determination based on the assumption that both variables correlate with vein diameter. Thus, we used the following equation to calculate the xylem construction cost of leaf veins:


$$\begin{array}{l} CC=\;\;\;\;\;\underset{i\;\;\;\;\;=\;\;1}{\overset{v}{\sum }}\;\pi \times d_{i}\times D_{i}, \end{array}$$ (2)

where *d*_i_ is the diameter of vein order *i* and *D*_*i*_ is the density of the same order.

### Leaf thickness

After measuring stomatal density and VLA, the same leaves were used to measure leaf thickness using freehand sections. The leaf sections were placed in water, and then mounted on slides and photographed. We measured the leaf thickness using Image J (http://rsbweb.nih.gov/ij/index.html) software.

### Data analysis

Independent *t*-tests were used to assess differences in leaf functional traits between EMH and TMH. Correlations between leaf traits were analysed with Pearson’s correlation coefficients. Principal component analysis (PCA) was used to analyse the correlations among the 18 plant functional traits and the distributions of the 27 species along the PCA axes by using ‘FactoMineR’ and ‘factoextra’ packages in R ver. 3.6.3 (R Core Team 2020). The phylogeny tree of the 27 studied species was generated from Phylomatic web site (http://phylodiversity.net/phylomatic/) by using the stored tree ‘zanne2014’ (Zanne *et al.* 2014). Phylogenetic ANOVA were used to test the differences in leaf traits between EMH and TMH by using the ‘geiger’ package (Pennell *et al.* 2014) in R. We used the linear descriptive analysis to select the most important variables for separating EMH and TMH by using ‘caret’ packages in R. SMATR v2.0 software was used to examine the differences in linear relationships between EMH and TMH (Warton *et al.* 2006).

## Results

We found that EMH had significantly higher mean leaf area, leaf thickness, stomatal density, *g*_max_, major vein diameter (including 1° VD, 2° VD and 3° VD) and stomatal number per vein length, but had lower mean major VLA, total VLA and the xylem construction cost of leaf veins than TMH (Table 2). The results of phylogenetic ANOVA followed the similar pattern with the results of normal one-way ANOVA; however, the differences in stomatal density and total VLA between EMH and TMH became marginally significant when considering the phylogeny relationships **[see ****Supporting Information—Table S1****]**.

**Table 2.** Leaf traits (mean ± SE) and the results of independent samples *t*-test between the 14 emergent aquatic and the 13 terrestrial monocotyledonous herb species (EMH and TMH), as well as 5 EMH_p_ and 8 TMH_p_, which belong to Poaceae. LA = leaf area, LT = leaf thickness, SD = stomatal density, SL = stomatal length, *g*_max_ = maximum modelled stomatal conductance, VLA = vein length per area, VD = vein diameter, CC= the xylem construction cost of leaf veins, SV = stomatal number per vein length.

		For all		Poaceae	
Trait	Unit	EMH	TMH	EMHp	TMHp
LA	cm^2^	77.1 ± 10.1	41.4 ± 9.5*	58.1 ± 11.7	25.6 ± 3.9^ns^
LT	μm	498.7 ± 107.8	216.1 ± 33.1*	397.3 ± 48.9	147.7 ± 6.2**
SD	no. mm^-2^	514.7 ± 102.0	254.8 ± 43.2*	724.8 ± 84.4	316.3 ± 43.1**
SL	μm	24.8 ± 2.0	25.4 ± 2.2^ns^	21.7 ± 1.5	24.2 ± 2.5^ns^
*g*_max_	molm^-2^s^-1^	0.47 ± 0.06	0.26 ± 0.03**	0.63 ± 0.06	0.30 ± 0.02**
1°VLA	mm mm^-2^	0.15 ± 0.06	0.07 ± 0.01^ns^	0.11 ± 0.01	0.09 ± 0.01^ns^
2°VLA	mm mm^-2^	0.76 ± 0.16	0.69 ± 0.11^ns^	1.09 ± 0.1	0.83 ± 0.12^ns^
3°VLA	mm mm^-2^	2.57 ± 0.55	6.29 ± 1.18**	3.49 ± 0.51	8.44 ± 1.07*
major VLA	mm mm^-2^	3.48 ± 0.72	7.05 ± 1.26*	4.69 ± 0.6	9.36 ± 1.15*
minor VLA	mm mm^-2^	3.44 ± 0.34	1.88 ± 0.52^ns^		
total VLA	mm mm^-2^	4.7 ± 0.54	7.49 ± 1.13*	4.69 ± 0.6	9.47 ± 1.09*
1°VD	μm	800.0 ± 160.7	484.5 ± 69.9	439.2 ± 93.6	388.7 ± 56.6^ns^
2°VD	μm	143.8 ± 21.8	73.9 ± 8.7**	94.1 ± 7.1	73.3 ± 4.7^ns^
3°VD	μm	46.1 ± 3.2	31.6 ± 4.3*	50.2 ± 2.4	28.7 ± 3.8**
minor VD	μm	21.7 ± 1.9	16.3 ± 2ns		
1 °CC	/	0.12 ± 0.02	0.09 ± 0.01	0.12 ± 0.02	0.09 ± 0.01^ns^
2 °CC	/	0.23 ± 0.04	0.15 ± 0.02*	0.3 ± 0.02	0.18 ± 0.02**
3 °CC	/	0.32 ± 0.06	0.5 ± 0.07^ns^	0.51 ± 0.05	0.63 ± 0.06^ns^
minor CC	/	0.25 ± 0.03	0.07 ± 0.01*		
total CC	/	0.76 ± 0.07	0.75 ± 0.08^ns^	0.93 ± 0.04	0.91 ± 0.06^ns^
*g*_max_/ total VLA	10^-4^molm^-1^s^-1^	1.10 ± 0.12	0.42 ± 0.07***	1.40 ± 0.10	0.36 ± 0.04***
SV	no.mm^-1^	106.3 ± 14.6	37.1 ± 6.4***	155.6 ± 11.0	33.5 ± 3.0***

*P < 0.05; **P < 0.01; ***P < 0.001; ns: *P* > 0.05.

**Figure 2.**
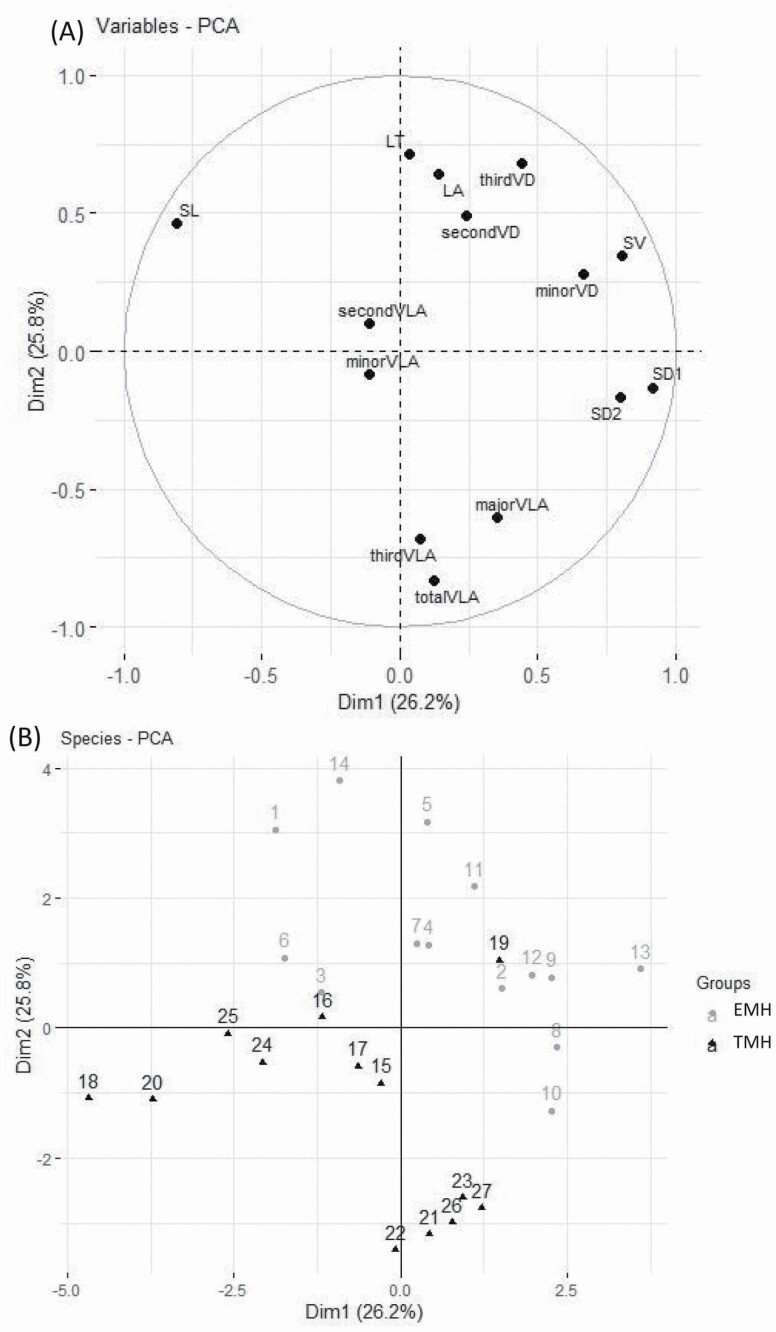
First two axes of the principal component analysis for the leaf functional traits and loading of the 27 species along the first two PC axes. The trait codes are as in Table 2 and species codes are as in Table 1.

Axis 1 and axis 2 of the PCA explained 42.6 and 21.2 % of the total variance, respectively. Axis 1 was loaded by stomatal density and VLA on the positive side and by leaf thickness and vein diameter on the negative side, whereas Axis 2 was loaded by stomatal number per vein length on the positive side (Fig. 2A). EMH and TMH can be separated from one another along axis 2, with EMH distributed on the positive side of axis 2 and TMH distributed on the negative side of axis 2 (Fig. 2B). There were 11 variables selected for the classification of the two groups with the linear discriminant analysis **[see ****Supporting Information—Table S2****]**. Within the 11 variables, stomatal number per vein length, leaf thickness, second vein diameter, third vein diameter and the xylem construction cost of the third leaf veins were the top five important variables.

**Figure 3.**
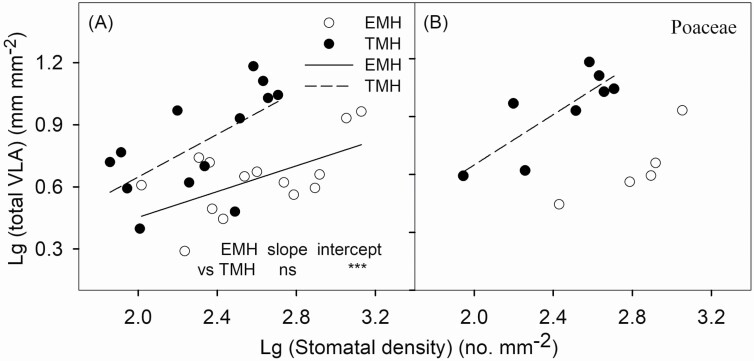
Stomatal density in relation to total vein length per area (total VLA) of terrestrial and emergent aquatic monocotyledonous herbs (TMH and EMH, respectively). Each point represents one species: (A) was for all species and (B) was for Poaceae species. The x-axis and y-axis are logarithmic. The correlations were statistically significant for both 14 EMH (total VLA = -0.17 + 0.31 × SD, *r*^2^ = 0.34*), 13 TMH (total VLA = -0.38 + 0.52 × SD, *r*^2^ = 0.40*) and 8 TMH_p_ (Poaceae species, total VLA = -0.65 + 0.65 × SD, *r*^2^ = 0.66*), but not significant for all the 27 species (*r*^2^ = 0.11, *P* = 0.08) and 5 EMH_p_ (*r*^2^ = 0.72, *P* = 0.07).*P < 0.05.

A significant and positive correlation between total VLA and stomatal density was found in both EMH and TMH (Fig. 3A; EMH, *r*^2^ = 0.34, *P* < 0.05; TMH, *r*^2^ = 0.40, *P* < 0.05), and the linear regression slope for these variables in EMH was significantly greater than that of TMH (Fig. 3A). Similarly, the relationships were also significant for TMH of Poaceae species (Fig. 3B; *r*^2^ = 0.66, *P* < 0.05), but not significant for EMH of Poaceae species (Fig. 3B; *r*^2^ = 0.72, *P* = 0.07).

**Figure 4.**
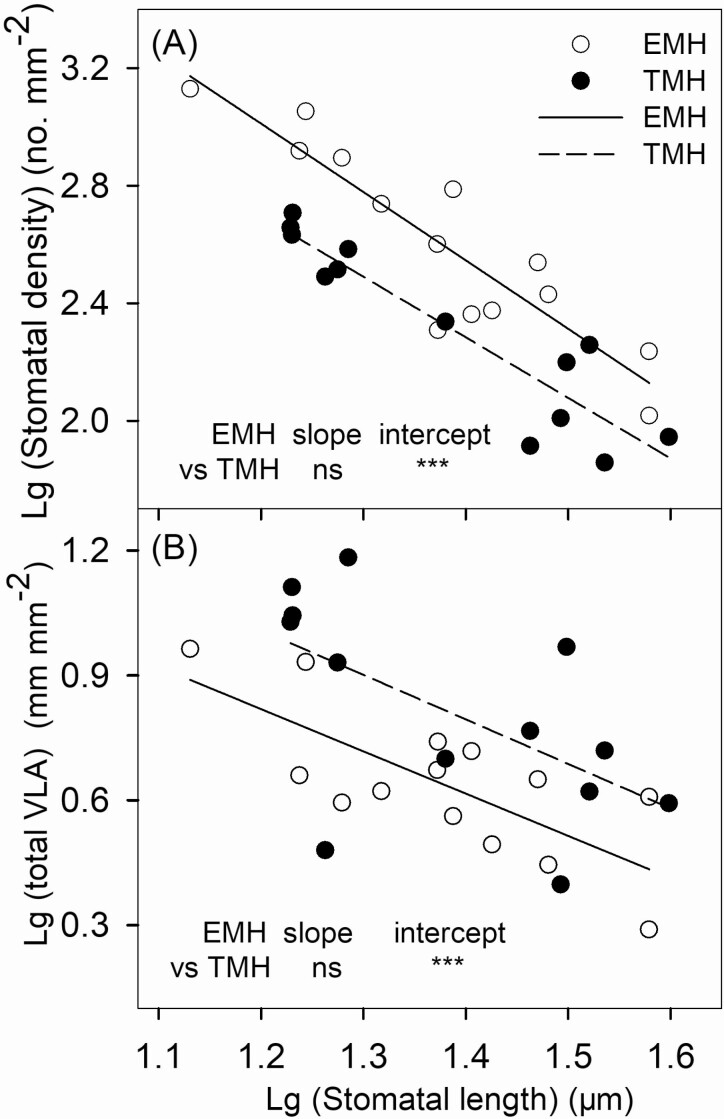
Stomatal length in relation to stomatal density (A) and total vein length per area (B) for 13 terrestrial and 14 emergent aquatic monocotyledonous herbs (TMH and EMH, respectively). Each point represents one species. The x-axis and y-axis are logarithmic. Correlations were statistically significant for both EMH and TMH in (A) (EMH, SD = 5.8–2.3 × SL, *r*^2^ = 0.82***; TMH, SD = 5.2–2.1 × SL, *r*^2^ = 0.85***) and (B) (EMH, total VLA = 2.0 - 1.0 × SL, *r*^2^ = 0.56**; TMH, total VLA = 2.3 - 1.1 × SL, *r*^2^ = 0.34*). *P < 0.05;**P < 0.01; ***P < 0.001; ns, *P* > 0.05.

Stomatal length was significantly and negatively correlated with stomatal density in both EMH and TMH (Fig. 4A; EMH, *r*^2^ = 0.82, *P* < 0.001; TMH, *r*^2^ = 0.85, *P* < 0.001), and the intercept of the regression line in EMH was significantly higher than that in TMH. The stomatal length was also significantly and negatively correlated with total VLA in both EMH and TMH (Fig. 4B; EMH, *r*^2^ = 0.56, *P* < 0.01; TMH, *r*^2^ = 0.34, *P* = 0.03), and the intercept of the regression line in EMH was significantly lower than that in TMH.

## Discussion

An important finding of this study is that the mean stomatal density of EMH was more than 2-fold that of TMH, whereas the total VLA of TMH was also significantly higher than that of EMH, which lead to the stomatal number per vein length of EMH was nearly 3-fold as that of TMH. For the Poaceae species only, the stomatal number per vein length in EMH was 5-fold as that of TMH. Compared with TMH, EMH appeared to have adapted to aquatic conditions via enlarged vein diameter and leaf thickness rather than increased VLA. However, we did not observe a positive linear correlation between VLA and stomatal density when the data of all species in both groups were pooled, although this relationship was found when the data of EMH and TMH were evaluated separately. Thus, our results showed that the coordination between leaf water supply and demand was environment-specific.

The results of the present study deepen the understanding of the coordination between stomatal density and VLA in emergent aquatic species. Although the positive linear correlation between stomatal density and VLA has been found in many terrestrial species under various conditions (Brodribb and Jordan 2011; Sun *et al.* 2014; Carins Murphy *et al.* 2016; Schneider *et al.* 2017; Zhao *et al.* 2017), we extended this correlation to the emergent aquatic herbs in this study. The intercept of the linear relationship between the stomatal density and total VLA in EMH was significantly lower than that of TMH, which indicated on a given value of stomatal density, TMH generally had higher VLA. The significant difference in the average stomatal number per vein length between the two groups also showed the difference in leaf water supply and demand coordination. EMH had significantly higher average stomatal density and lower average total VLA than TMH, which might be mainly because EMH had larger vein diameters (Table 2).

A comparison of the specific changes in coordination between leaf water supply and demand of different plants could help explain the changes in leaf water-use strategies. Vein density and diameter determine the water transport efficiency of the leaves and reflects transpirational characteristics (Boyce 2009; Sack and Scoffoni 2013). In aquatic environments, a low VLA reduces mesophyll displacement inside leaves (Carins Murphy *et al*. 2014). Higher major vein diameter might results in larger vessels within these veins, thereby providing greater maximum hydraulic conductivity in EMH species. EMH species evolved higher stomatal density to match this greater flow and theoretical *g*_max_. Furthermore, these leaves are rarely exposed to substrate water deficit so evolving very large major vein diameters and vessels would not be maladaptive because these veins would be rarely exposed to embolism or cell collapse. On the contrary, TMH usually endure more drought stress in the dry season than EMH. Consequently, TMH invest more energy to build denser veins with a smaller diameter in their leaves. Actually, species in drier areas do have higher VLA (Sack and Scoffoni 2013). In this study, we also found that the TMH have significantly higher VLA than EMH species, which might be because of TMH species are more prone to xylem embolism induced by drought. Increasing vein density may provide increasingly redundant pathways for water flow (Scoffoni *et al.* 2017; Scoffoni and Sack 2017).

The emergent aquatic environment also deeply influenced the stomatal traits of EMH, as the average stomatal density of them was more than 2-fold that of TMH, but the stomatal length of both groups was not significantly different. Monocots have distinctly lower leaf vein densities than other angiosperm subclades (Roddy *et al.* 2013; de Boer *et al.* 2016), which indicated that monocots may experience less evolutionary pressure to increase leaf gas exchange capacity despite having both leaf sides available to allocate to stomata (Rudall *et al.* 2017; Haworth *et al.* 2018). Alternatively, monocots do experience selection for increased gas exchange capacity, but because of C4 photosynthetic pathway, the scaling of VLA and the maximal photosynthesis is different than in C3 plants (Sack and Scoffoni 2013). Hence, the competitive advantage of spatially optimal location of leaf epidermal area to stomata could be negated by specific growth conditions in relation to leaf hydraulics and leaf morphology (de Boer *et al.* 2016). In this study, when the data of both the groups were pooled, the stomatal length and total VLA were still significantly and negatively correlated (Fig. 4B; *r*^2^ = 0.34, *P* < 0.01), although the intercept of the regression line in EMH was significantly higher than that in TMH. Previous studies had also reported this relationship in other species (Zhang *et al.* 2012; Zhao *et al.* 2016).

## Conclusions

The emergent aquatic herbs exhibited considerable differences in their water-related functional traits when compared with terrestrial herbs, with the former having greater water transport capacity and stomatal conductance potential. Although a correlation between stomatal density and total vein density was found in each group, this correlation became non-significant when the data from both the groups were pooled. Our results showed that different water conditions modified the coordination between leaf veins and stomatal traits of emergent aquatic and terrestrial plants. The present study also provided new evidence that supporting the hypothesis of a leaf water supply and demand hypothesis.

## Supporting Information

The following supporting information is available in the online version of this article—

**Table S1.** The results of the one-way ANOVA test and phylogenetic ANOVA.

**Table S2.** The results of the Linear Discriminant Analysis (LDA) analysis.

**Figure S1.** Phylogeny tree of the 27 studied species.

plaa047_suppl_Supplementary_Material

plaa047_suppl_Supplementary_Data

## Notes

**Form & Function. Chief Editor**: Kate McCulloh
