# Supplementary material for: Difference between emergent aquatic and terrestrial monocotyledonous herbs in relation to the coordination of leaf stomata with vein traits
Source: AoB Plants. 2020 Sep 11;12(5):plaa047. doi: 10.1093/aobpla/plaa047 (PMC7750939; doi:10.1093/aobpla/plaa047)
Supplement: plaa047_suppl_Supplementary_Material [file plaa047_suppl_supplementary_material.docx]

Table S1 The results of one-way ANOVA test and Phylogenetic ANOVA. The significant results were in bold. The traits codes are as in Table 2.

| Trait | Unit | F | One-way ANOVA | Phylogenetic ANOVA |
| --- | --- | --- | --- | --- |
| LA | cm^2^ | 6.628 | **0.016** | **0.020** |
| LT | μm | 5.888 | **0.023** | **0.039** |
| SD | no. mm^-2^ | 5.210 | **0.031** | 0.059 |
| SL | μm | 0.039 | 0.845 | 0.863 |
| *g*_max_ | molm^-2^s^-1^ | 9.545 | **0.005** | **0.020** |
| 1°VLA | mm mm^-2^ | 1.420 | 0.244 | 0.392 |
| 2°VLA | mm mm^-2^ | 0.109 | 0.744 | 0.784 |
| 3°VLA | mm mm^-2^ | 8.557 | **0.007** | **0.020** |
| major VLA | mm mm^-2^ | 6.354 | **0.018** | **0.020** |
| total VLA | mm mm^-2^ | 5.212 | **0.031** | 0.059 |
| 1°VD | μm | 3.072 | 0.092 | 0.098 |
| 2°VD | μm | 8.345 | **0.008** | **0.020** |
| 3°VD | μm | 7.614 | **0.010** | **0.020** |
| 1°CC | / | 2.726 | 0.111 | 0.157 |
| 2°CC | / | 4.115 | 0.053 | 0.059 |
| 3°CC | / | 3.868 | 0.060 | 0.078 |
| total CC | / | 0.012 | 0.913 | 0.922 |
| SV | no.mm^-1^ | 17.85 | **<0.001** | **0.020** |

Table S2 The results of the Linear Discriminant Analysis (LDA) analysis. The 11 selected variables by the model were shown, and the top five variables were in bold. The trait codes are as in Table 2.

| Variables | Accuracy | Kappa | Accuracy SD | Kappa SD |
| --- | --- | --- | --- | --- |
| 2°VD  SV  LT  3°VD  3°CC  SD  g_max_  LA  2°CC  1°CC  1°VD | **0.90** | **0.80** | **0.21** | **0.42** |

Figure S1 Phylogeny tree of the 27 studied species.
